# Sample Preparation to Determine Pharmaceutical and Personal Care Products in an All-Water Matrix: Solid Phase Extraction

**DOI:** 10.3390/molecules25215204

**Published:** 2020-11-09

**Authors:** Daniele Sadutto, Yolanda Picó

**Affiliations:** Food and Environmental Safety Research Group, Desertification Research Centre—CIDE (CSIC-UV-GV), University of Valencia (SAMA-UV), Moncada-Naquera Road, Km 4.5, 46113 Moncada, Spain

**Keywords:** pharmaceuticals and personal care products, isolation, concentration, solid-phase extraction, cartridges, disks, online, dispersive liquid-liquid microextraction, water samples

## Abstract

Pharmaceuticals and personal care products (PPCPs) are abundantly used by people, and some of them are excreted unaltered or as metabolites through urine, with the sewage being the most important source to their release to the environment. These compounds are in almost all types of water (wastewater, surface water, groundwater, etc.) at concentrations ranging from ng/L to µg/L. The isolation and concentration of the PPCPs from water achieves the appropriate sensitivity. This step is mostly based on solid-phase extraction (SPE) but also includes other approaches (dispersive liquid-liquid microextraction (DLLME), buckypaper, SPE using multicartridges, etc.). In this review article, we aim to discuss the procedures employed to extract PPCPs from any type of water sample prior to their determination via an instrumental analytical technique. Furthermore, we put forward not only the merits of the different methods available but also a number of inconsistencies, divergences, weaknesses and disadvantages of the procedures found in literature, as well as the systems proposed to overcome them and to improve the methodology. Environmental applications of the developed techniques are also discussed. The pressing need for new analytical innovations, emerging trends and future prospects was also considered.

## 1. Introduction

The production and consumption of pharmaceutical and personal care products (PPCPs) is considered an important environmental risk [1,2,3,4]. In the last decades, the occurrence of these compounds in nature increased, as described in a number of studies [5,6,7]. PPCPs can be detected as the active substance, with an unaltered chemical structure, or as a metabolite or a degradation product produced by human and environmental enzymatic activity [8], weather conditions, wastewater treatments [9] and by chemical-physical properties of matrices. There are many reasons for the increasing occurrence of these compounds in different environmental compartments, e.g., their intensive use in farms and aquaculture [10,11,12] or their inefficient removal from wastewater treatment plants [13,14]. The latter explains why PPCPs used and excreted at home or in hospital can ultimately be released into the environment. Another important source of contamination by PPCPs is industrial waste [15], which is not always processed in the correct form. Furthermore, treated wastewaters are reused for agriculture activity, especially in arid regions [16], contributing to the spread of PPCPs in more matrices, such as soil, wild animals, vegetation, and even food crops.

These considerations on the sources of PPCPs shows the key role that the analysis of water plays to fight against contamination. In fact, water—the most affected environmental compartment—may be considered as a mirror of the pollution status of an area, and also a scarce resource that must be preserved with optimal quality and zero pollution. In addition, water contaminants, depending on their physicochemical properties, may also (bio)accumulate in sediments and biota, consequently harming human health [17]. Therefore, it was considered a relevant vehicle to different environmental compartments.

Determining and quantifying PPCPs in different types of water provides considerable interesting information not only related to pollution status. For example, the analysis of wastewater samples, divided into influent and effluent waters, could offer information on the PPCPs consumption of a community, estimate the wastewater treatment plant efficiency and establish the most recalcitrant compounds difficult to eliminate. River, lake, and seawater samples could give us an idea of the more persistence substances. The detection in irrigation channels could identify food quality issues. In addition, drinking water is certainly another matrix that should be monitored to assess the potential risks on human and animal health for long-term use [18,19].

For an accurate analysis of PPCPs in different types of water, it is fundamental to consider all water characteristics that could influence recovery of the contaminants. The pH could affect the structure of molecule, promote ionization according to pKa, or activate a prodrug with a change of structure. Many substances were thermolabile and photosensitive, for this reason, sample temperature must always be considered. The salinity of water could increase or decrease extraction efficiency due to different ionic strengths of the media, or the formation of molecular complexes between PPCPs and multivalent metal cations present in the samples that are soluble in water [20]. Other water components have also a strong influence on the PPCPs recovery because are responsible for degradation and/or metabolism. A large range of different metabolites can be formed depending on the specific enzymatic activities, presence of fulvic and humic acids, microorganisms, etc. [21,22,23].

In addition to the matrix, it is also important to consider the structural variability of PPCPs, designed to interact with specific targets. The presence of distinct functional groups (such as esters, carboxylic acid, ketones, amides, etc.) or the existence of nucleophile/electrophile substituents contribute to all chemical-physical characteristics of each active substance. Influencing stability, reactivity, and solubility in water are all parameters that need to be considered before a sample’s preparation for analysis. Despite the variability in the PPCPs’ chemical structures, for most laboratories specialized in the analysis of these compounds, the use of multiresidue methods is very attractive because not only attains a reduction of cost and time, but also offers global patterns of contamination with only one analysis. Moreover, these methods easily facilitate an eco-friendly analysis with decreases in waste. In these multiresidue methods, the sample preparation becomes the heart of analysis that influences any other procedural steps from sample collection and storage to the specific instruments selected for final quantification (high performance liquid chromatography-mass spectrometry (HPLC-MS), gas chromatography-mass spectrometry (GC-MS), etc.). The choice of a liquid or gas chromatography (LC or GC), to analyze the final extract, is guided by the analytes’ polarity. Generally, compounds with polar characteristics are more suitable for LC, and those with non-polar properties are more amenable to GC; most PPCPs are polar or moderately polar [24]. Surprisingly, some contemporary review articles either cover broader aspects of environmental analysis [25,26,27], or focus on a particular type of extraction process (e.g., microextraction, use of nanomaterials, magnetic, ultrasonic, etc.) [28,29,30] but do not cover the entire sample preparation.

Therefore, the goal of this review was to critically analyze the status of sample preparation to determine PPCPs in an all-water matrix. Each step of sample preparation, including all analytical variants (conventional and innovative methods) used to detect these contaminants were considered. Furthermore, each sample preparation method was critically analyzed, highlighting advantages and disadvantages. This review performs an examination of all studies published from January 2018 to May 2020. The search was conducted on the database Scopus (Elsevier), with two different inputs: “extraction pharmaceutical environmental”/“extraction personal care products environmental” and “extraction pharmaceutical water”/“extraction personal care products water”. More than one thousand one hundred works have been viewed. The selection criteria to choose the studies were based on (i) the presence at least of 10 PPCPs to include attractive multiresidue methods; and (ii) water compartmentation, in all variants (wastewater, rivers, irrigation channels, lakes, drinking water, seas, urban storms, swimming pools and thermal water), was chosen. In addition, some reviews outside the interval of time were also chosen.

## 2. Extraction and Clean-Up of PPCPs in Water

**Figure 1:**
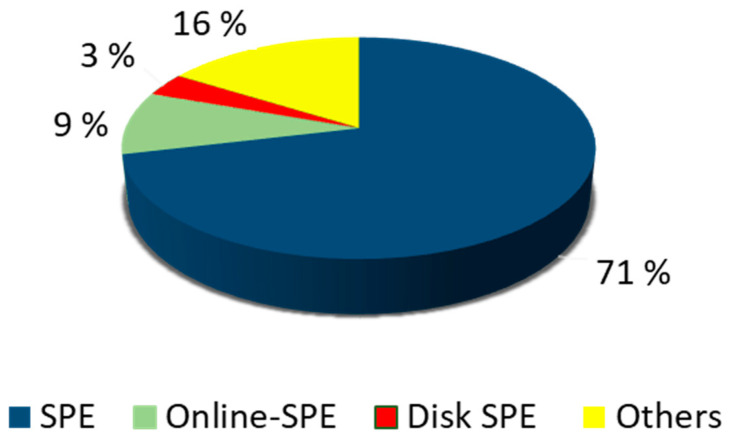
Pharmaceuticals and personal care products (PPCPs) extraction procedures according to the percentage of studies that applied them. SPE: solid-phase extraction.

PPCPs are organic compounds, and traditionally this type of compound has been extracted by solid-phase extraction (SPE). This technique was commercialized in the late 1970s and rapidly replaced the liquid-liquid extraction that was previously used [31]. Table 1 and Figure 1 show the analytical methods applied to extract PPCPs in water. The most common were still based on SPE in all possible variants (cartridge, disk, offline, online, etc.). The classic SPE process (cartridges offline) was used in 71.3% of the studies, the online version was employed in the determination in 9.2%, and disks were utilized instead of cartridges in 3.2%.

Only 16% of the studies use other types of methods, such as direct injection, dispersive liquid-liquid extraction (DLLME) (based on liquid-liquid extraction), polyether sulfone microextraction (PES) or buckypaper devices. It is important to note here that many of the methods classified as “other” are based on the basic principles of SPE, but using new phases or formats.

### 2.1. SPE

This technique involved the use of a small amount of sorbent (commonly hundreds of mg) in a cartridge or syringe barrel. After activation of the sorbent, a water sample of hundreds of mL was passed through the sorbent, which retained the analytes of interest (in this case PPCPs) whereas the water was discarded. Then, the analytes retained in the sorbent were eluted using a few mL of organic solvent. This technique has some advantages, such as the minor investment in reagent and materials, and rapidity.

#### 2.1.1. Sorbents and Formats

There are different marketed sorbents that work principally with two distinct separation mechanisms: classical reversed phase chromatography (RP) or ion exchange chromatography (IC). Characteristics of these sorbents are summarized in Table 2. The sorbents used in RP are mostly of a polymeric nature and could be used for a large spectrum of PPCPs, including acidic, basic, and neutral compounds. RP sorbent was applied in 85% of SPE approaches (see Table 1). This is attributable to its ability to retain a wide range of different polarity compounds, a relevant characteristic for a multiresidue method that includes different chemical classes. Of these, two-thirds had a stationary phase characterized by polymeric sorbent that contained vinylpyrrolidone (Oasis^®^ HLB, Strata^®^X and Cleanert^®^ PEP). Another polymeric sorbent applied was marked by the presence of polystyrene-divinylbenzene (Isolute^®^ ENV+, Chromabond^®^ HR-X and Bond ElutTM ENV) that was applied to polar analytes, or the presence of octadecyl endcapped silica RP (Supelclean™ LC-18 SPE) that was used for nonpolar to moderately polar analytes from aqueous samples.

The IC sorbents were used in the so-called mixed-mode cartridges that combine the polymeric sorbent with an ionic exchanger that could be weak or strong. The IC can be of cations or anions, and this affects the target specificity. Cationic exchange sorbents (weak or strong) were designed to extract basic PPCPs, and anionic exchange sorbent (weak or strong) were to extract the acidic ones. However, weak polymeric cationic-exchangers (Oasis^®^ MCX, Strata^®^X-CW or X-Drug B and UCT XTRACT^®^ XRDAH) were the most prevalent.

Different studies applied attractive modifications to these traditional cartridges, to obtain the best recoveries for a large spectrum of compounds. Zhu et al. [52] developed, characterized and tested a hydrophilic resin based on poly(*N*-vinyl pyrrolidone-co-divinylbenzene) (NVP-co-DVB) that improved the average absolute recovery for 44 PPCPs, with respect to the use of HLB sorbents. Alternatively, Caban et al. [62] studied the modification of the columns through the application of additional sorbents on top of a polymeric HLB column to improve the SPE of 15 analytes (pharmaceuticals and estrogens) from water. PSAs (Primary and Secondary Amines) and alumina retained matrix components (e.g., humic and fulvic acids) without decreasing the analyte recovery. The solution was named triple-sorbents SPE. They were applied in order to reduce matrix effect. Similarly, Gago-Ferrero et al. [53] mixed four SPE materials simultaneously in an in-house cartridge. These materials included classical RP and ion mixed-mode sorbents (Oasis HLB, Isolute ENV+, Strata-X-AW and Strata-X-CV). Salas et al. [102] combined anionic and cationic exchange sorbents in the same cartridge to extract basic and acidic pharmaceuticals simultaneously. These minor improvements in the SPE procedure (without any relevant cost increment or enlargement of the procedure) can produce an important effect on the quantification of PPCPs, and increase the reliability and reproducibility of the results.

The format of the cartridge and the volume of samples that pass through the cartridge were other elements to take into consideration. The sorbent weight (mg), capacity (mL) and pore size (µm) can influence the efficiency of the columns. These parameters have a key role on the surface area, on which analytes interacted. The amount of sorbent ranged from 60 to 600 mg, and the most used was a quantity of 200 mg. The capacities most used were 3 and 6 mL. There is a global consensus on this point.

In SPE, the volume of water processed generally involves hundreds of milliliters. The capacity to detect lower amounts is one of the advantages of SPE. The matrix effect (ME) could be an element to take in consideration in the choice of volume because it is directly proportional to volume as well as the organic matter content of the water; in this case, clogging of the cartridge slows the process too much. For this reason, some studies chose different volumes of water (depending on its characteristics) with the same method. For example, influent wastewater samples were generally analyzed with smaller volumes compared to effluent samples [46,48].

**Figure 2:**
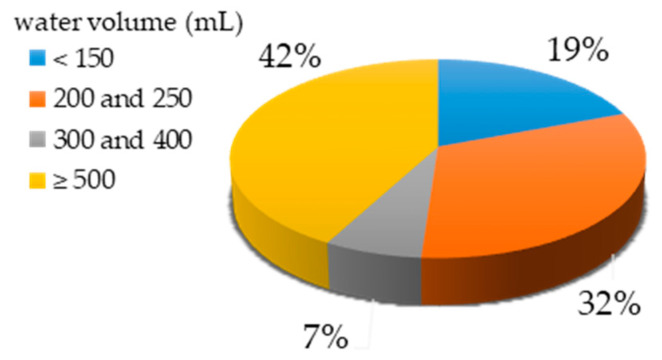
Percentage of studies according to water volume.

Figure 2 compares the various volumes that were selected in the different studies. There are two ranges of volumes commonly chosen—between 200 and 250 mL (32% of the studies) and in interval ≥500 mL (42% of works). The volumes commonly used were 200, 250, 500 and 1000 mL. The latter is most commonly selected, but mainly for surface and drinking water.

#### 2.1.2. SPE Activation, Washing and Elution

In SPE, the first step is the conditioning of the sorbent in order to favor the interaction of the sorbent with the analytes by a mechanism namely “solvation”. RF and IC sorbents are usually activated by filling the column two or three times with a solvent miscible with water (e.g., methanol, acetonitrile) followed by the solvent in which the analyte is dissolved (pure matrix, e.g., water, buffer). Methanol and water are most frequent solvents to activate cartridges. Water used for activation can be pH-adjusted or spiked with salt, counter ions or metal sequestrators (sodium acetate buffer [106], monopotassium phosphate [95], sodium dodecyl sulphate [57], etc.) to promote several types of interactions with compounds.

Once the sample passed through the sorbent, another important step is to remove the impurities retained on the SPE packing. For this reason, a wash solution strong enough to remove these impurities, but weak enough to leave the analytes of interest, is passed through the cartridge. These solutions are water, pH-adjusted water, or in a few cases methanol-water (5:95, *v*/*v*). The wash was followed by cartridges air-drying by vacuum or pressure to remove the remaining water, although in few cases, the cartridges can be dried with nitrogen instead of air [37,48,55,61,63].

The RP SPE is the mechanism more commonly used to extract contaminants from water samples, as described previously. The hydrophobic or non-polar interactions between sorbent functional groups and analytes must be destroyed with an organic solvent or solvent combination of enough non-polar character. The most used elution solvents are methanol and acetonitrile. The pH modification during elution can improve recovery if the analyte is ionizable and the eluent favors its ionic form, and basic and acidic compounds become more polar [107].

**Figure 3:**
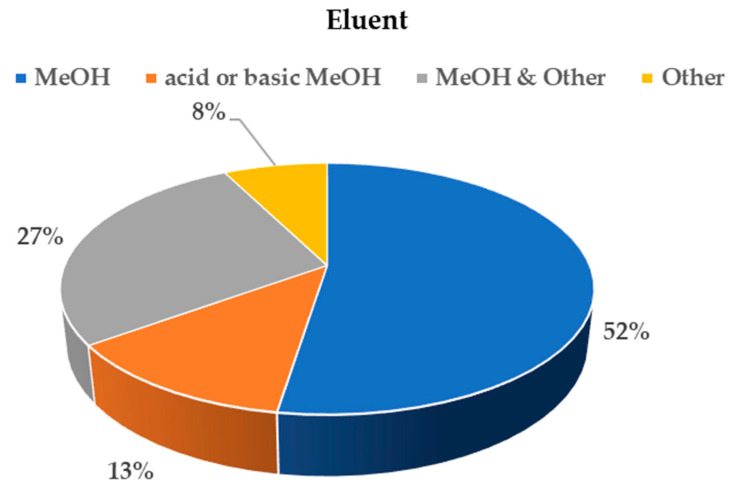
Percentage of studies according to the type of eluent used.

Figure 3 shows the eluents most frequently applied with HLB sorbents. Only methanol was used in more than half of methods, with acid or basic pH adjustment in 13%, and with a mix of other solvents or followed by a second elution with different solvent, for example, acetonitrile, dichloromethane and acetone, in 27%. Only 7% of the methods presented an eluent mixes (mostly acetonitrile and acetone) without methanol. In the case of weak cationic-exchange sorbents, the eluent was a basic solution with NH_4_OH in methanol or acetonitrile.

It is fundamental to consider not only the elution but also all steps that follow the elution. Once the PPCPs have been eluted, the extract is evaporated under a gentle stream of nitrogen in order to concentrate the analytes. The temperature used in this evaporation step is an important parameter that could affect the molecular stability of PPCPs. However, this temperature was not always reported in the studies, even though it can be responsible for the degradation of some PPCPs. The temperature range was between 25 and 50 °C, and the average was 39 °C.

The dried extracts are reconstituted with one or more solvents. If the compounds are determined by HPLC-MS, the mixture of solvents is usually comparable in composition to the initial mobile phase. Again, the reconstituting solvent more commonly used was the methanol-water mix. The most common volumes used for reconstitution were 0.5 and 1 mL. In the case of GC analysis, the addition of a derivatization reagent was often required because PPCPs are mostly polar and/or thermolabile, and these compounds are not amenable for GC analysis without increasing the stability of analytes.

Lastly, the final extract is filtrated before the analysis. Different syringe filters were reported, such as nylon, PVDF (polyvinylidene fluoride), PTFE, RC, GHP, PP, etc. The size used were 0.20 and 0.22 μm. This procedure was an ulterior clean-up to remove the particles with a large size to optimize analysis, for example by reducing the presence of obstruction phenomena in column. For this step, it was fundamental to remember which combinations (solvent-filter) are safe and which are corrosive.

#### 2.1.3. Water Sample Pretreatment before SPE

Regarding the characteristics of the water sample, the preservation of its integrity from the sampling point to the moment of the analysis was vital. To this end, in many cases, the water samples were spiked by preservative compounds or solutions. As described in the U.S. Library of Medicine (2017), a pharmaceutical preservative is referred to as “substances added to pharmaceutical preparations to protect them from chemical change or microbial action” [108]. In the same way, these preservatives can be added to the water sample to avoid the degradation of the PPCPs present in the sample. Preservatives can be natural or synthetic compounds and included buffers, bulking agents, chelating agents, antioxidants, antimicrobial agents, surfactants, etc. ” [108,109]. The most used was EDTA—a chelating agent thanks to its four carboxyl groups and two nitrogen atoms that can form stable complexes with cations—which is able to improve the extraction efficiency of certain pharmaceuticals that also form complexes with metals, such as antibiotics, because they sequestrate the metals of the solution, liberating the PPCPs and increasing their recovery [110]. Generally, EDTA was added in the samples to a final concentration of 0.1% (1.000 g/L) or 0.05% (0.500 g/L), but it could reach a final concentration of 0.2, 2 or even 5%. Other preservatives used were antimicrobials, such as formaldehyde, NaCl, sodium azide and citric acid, and/or antioxidants, such as ascorbic acid and sodium thiosulfate. These antioxidants reduced any residual chlorine, chloramine and ozone that had been used as a disinfectant because they could react with some antibiotics [97]. Furthermore, more than half of the methods adjusted the pH of the sample to prevent degradation, ionization phenomena, or to achieve the optimum value for the extraction. For about 70% of methods, pH was adjusted between 2 and 3 units. In a few cases this adjustment was between 3.5 and 7. It was rarely adjusted by 9 and 10 units.

To ensure the proper quantification of the analytes is always very important. Therefore, samples were spiked by a solution of internal standard (IS) in more than 83% of the studies to obtain more reliable results, taking matrix effects into consideration. It was not always possible to use an isotopic reference for every compound, because the cost of the ISs are high, and are not available for some of the target analytes [57].

Another pretreatment widely used in water sample preparation was filtration, the role of which was to remove suspended substances, such as suspended particles, colloids and microorganisms from samples to prevent obstruction of the SPE cartridges or significant interferences in subsequent treatment processes [74]. In various studies, it was easy to find filters constituted of different materials, such as paper, nylon, PVDF (polyvinylidene fluoride) and cellulose membrane. The most frequently used to monitor the water pollution was a glass microfiber filter. The mesh filter range was from 0.20 to 1.60 μm; the size most commonly used was 0.45 μm. Sometimes filters with different sizes were coupled to remove particles at different levels. Centrifugation was an alternative to filtration, with the same goal but much less used [32]. In this case, the mass deposited on the bottom was removed and analysis was focused on supernatant.

### 2.2. Online SPE

SPE can be used offline (independently from the further chromatographic analysis) or online (directly connected to the chromatographic system). However, there are few difference in the components of the techniques between the two formats, with the exception of the valves system used to connect SPE online with the determination technique (commonly any type of HPLC-MS). In both, the main factors that affected the results of these technique were the formats and sorbents of stationary phase (cartridge) and the solvent(s) used for activation, washing and elution. Online SPE can be coupled to both, LC or GC. However, the preferred technique is online SPE liquid chromatography tandem mass spectrometry (SPE-LC-MS/MS), PPCPs and the SPE eluents are much more compatible with the mobile phase of LC than with that of GC.

**Figure 4:**
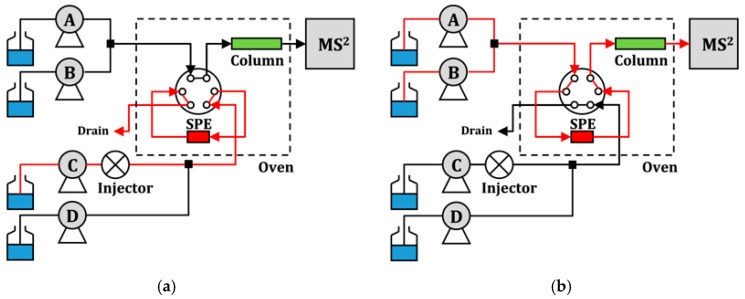
Configuration of the online SPE liquid chromatography tandem mass spectrometry (SPE-LC-MS/MS) system. I. separation column equilibration (pump A and B); II. sample loading and trapping (pump C); III. SPE desorption (pump A and B) and cleaning (pump D). Reproduced from [68] with permission from Elsevier. (**a**) Sample loading (Pump A, B, and C: On); (**b**) Sample analysis (Pump A, B, and D: On).

To be functional, online systems require the use of 6 or 10 port valves to automate extraction and connection to the instrument. A schematic illustration of the analytical system is presented in Figure 4. The most common are home-made devices, but there are also commercial systems such as an automated sample processor [82]. These devices can be personalized and adapted to the particular analysis, for example, to obtain online cross, which allows the automatic cross-utilization of two SPE columns to speed up the analysis of pharmaceuticals in different water samples [40].

The columns for online-SPE mode were characterized by similar polymeric RP sorbents. The main differences, with offline-SPE, were the length of columns (generally 1–2 cm). OASIS HLB (2.1 × 30 mm, 10 μm) [40,50,78], MAYI-ODS (10 mm × 2.0 mm, 50 μm) [68], and PLRP columns [39,82] have been the most widely reported. However, sorbents based on alternative mechanisms, such as the so-called TurboFlow™, which mixes size exclusion chromatography with reversed sorbents, has also been reported to determine pharmaceuticals [46]. To this end, three TurboFlow™ columns (TFC) connected in series were used, i.e., Cyclone P–C18-P XL–Cyclone MAX in order to achieve a proper extraction and clean-up.

The online SPE systems present the advantage to provide automatic and efficient sample loading, clean-up, desorption, separation, and detection at the same time, to reduce the sample volume, save time and solvents, prevent sample contamination and PPCP loss, and improve the method performance. The reduction in sample volume that could decrease sensitivity is normally compensated for by the increase in sensitivity as all the analyte retained in the sorbent passes to the chromatographic column.

The disadvantage of these methods is that they are not very versatile to be adapted to different types and conditions of analysis, because sample pH, injection volume, and valve-switching time needs to be very carefully optimized to ensure appropriate method performance for target PPCPs [39]. Once established, they are more suitable for routine analysis.

### 2.3. SPE Disk

The disks are a variant of cartridges that follow the same principle to retain an elute PPCPs. The disks have a higher diameter (commonly ca. 45 mm) and low height (a few millimeters). This format attempts to address several disadvantages of the cartridges, such as plugging due to the suspended particulate matter, high back-pressure that reduces flow rates, and improve retention kinetics of the analytes by using lower particle size. Generally, it was used with higher volumes of samples than SPE. The passage of the sample was much quicker (up to 100 mL/min). Moreover, the use of disks was advantageous for handling dirty samples. Only four studies used this approach (Table 1). There are disposable disks of many types of sorbents: C18, hydrophilic divinylbenzene (DVB), HLB or carbon.

Hydrophilic divinylbenzene (DVB) disks have been compared with Oasis HLB cartridges. Although the most apolar analytes (LogP ≥ 4) attained higher process efficiencies following Speedisk extraction, it could be noticed that in general, process efficiency was lower than for Oasis HLB extraction: 16 versus 59 analytes having a process efficiency >60% for Speedisk and Oasis HLB, respectively [71]. However, this study did not compare the same type of sorbent in both formats. Kafeenah et al. [103] did compare both formats using HLB sorbent. The method using disk SPE was better in terms of recovery, sensitivity, rapidness, and matrix effect, compared to the cartridge method.

The combination of different disks in order to improve recoveries has also been tested for GC-MS amenable analytes using, in sequence, a glass microfiber disk (GMF 150, 47 mm, Whatman), a styrene-divinylbenzene disk (Empore™ SDB-XD, 47 mm), and an active carbon disk (Empore™ AC, 47 mm) [99]. However, the same study recommended the use PS-2 and AC-2 Sep-Pak short cartridges for compounds analyzed by HPLC-MS.

The main disadvantages, as evidenced in the studies, are related with highest waste of samples and reagents used to active, wash and elute the sorbent.

### 2.4. Other Extraction Approaches

Other approaches have been reported to extract the PPCPs from water, and even though they are not as used as SPE, can be advantageous for some applications. These approaches are commonly focused on more environmentally friendly alternatives that reduce the use of materials, organic solvents and reagents; the so-called green chemistry. The simplest process is direct injection without any pre-concentration steps [78,83]. This was possible thanks to the excellent sensitivity of the HPLC-MS/MS. Botero-Coy et al. [83] included a simple dilution with water (×5) in order to reduce the matrix complexity. The most important problem in this method is its high matrix effects.

The microextractions, both solid and liquid, are an attractive alternative to SPE. The Dispersive Liquid-liquid Microextraction (DLLME) [37,47] has benefits related to a quick, easy cleaning and highly efficient pre-concentration procedure. Moreover, the sample volume required was very small, reducing the wastes. Two solvents were used, a dispersant and an extractor. The dispersant must be soluble in water and in the extractor, and the choice was methanol. As extractor (few microliters) both, the most traditional, chloroform, and the most recently introduced ionic liquids (1-Hexyl-3-methylimidazolium hexafluorophosphate) have been reported. The most important problem of this technique to determine PPCPs is that it is more efficient for non-polar compounds. Other approaches applied were the solid phase microextraction (SPME), which is attractive, because it enables extraction and clean-up in only one step, eliminating the problems associated with extensive use of solvents and equipment, since the analytes retained in the fiber can be thermally desorbed in the GC injector [111]. Another advantage of this technique is that the adsorbed analyte can be derivatized on-fiber, to transform it into a more volatile compound in order to make it more GC-MS amenable. Recently, this on-fiber derivatization and on-line thermal desorption has been applied for the extraction of 17 mL of water sample for the simultaneous determination of 22 pharmaceuticals and personal care products, including three transformation products, in sewage [89]. The fiber used was 2-cm long, 50/30-µm thick Divinylbenzene/Carboxen/Polydimethylsiloxane (DVB/CAR/PDMS). This method has the advantage of avoiding the use of organic solvent, since analytes are directly desorbed in the GC injector. SPME fiber can also be desorbed with a few mL of organic solvent to analyze the PPCPs by HPLC-MS. As in the method reported by Mijangos et al. [49], preconcentrated pharmaceuticals and personal care products in a disposable and low cost polyethersulfone (PES) sorbent are further desorbed in methanol.

Other approaches are based on testing new phases consisted of nanomaterials as more efficient sorbents. Tomai et al. [93] performed an SPE with oxidized buckypaper (BP) for Stir-Disc. The aim was to propose an SPE extraction device which combined the properties of carbon nanotubes and magnetic stirring with the main advantages of disk SPE. The concept was the same as classic SPE, but in this case the extraction device was immersed into the aqueous sample and left under magnetic stirring to permit analyte absorption on a BP membrane.

The last approach was the use of a PASSIL sampler [79]. The acronym PASSIL describes a device constituted of two PES membranes impregnated with an ionic liquid. After passive sampling, the receiving phase was eluted from the membranes and dissolved with acetonitrile.

## 3. Environmental Applications

**Figure 5:**
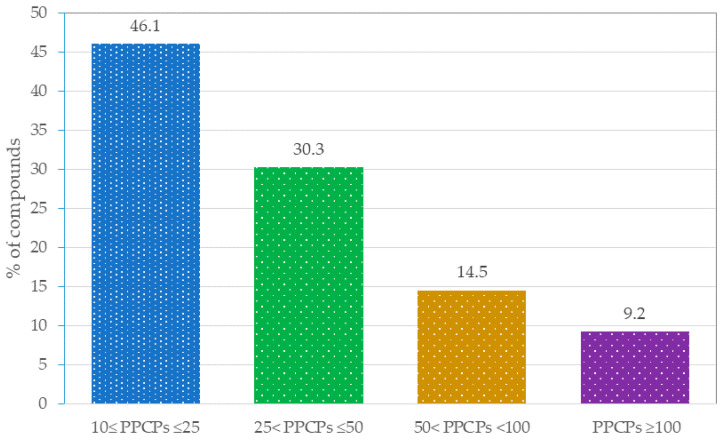
Percentage of studies according to the number of PPCPs analyzed.

Seventy-six studies have been selected (Table 1). Nine studies proposed the application of two different methods or approaches for sample preparation. In many cases, the method was applied to various aqueous matrices. The average number of PPCPs detected for each method was forty-one. The average number of PPCPs included in the studies reviewed increased over time, which may be justified by the growing interest in using methods that include as many substances as possible in the same analysis. In the studies published in 2018, the average number of PPCPs included was 34; in 2019 the average was 38 and up to May 2020 it was 60. In the Figure 5, the different studies are classified according to the number of compounds detected. Forty-six percent of studies covered between 10 and 25 PPCPs. The second range (between 26 and 50) included 30% of studies. Fifteen percent showed a 50 < PPCPs < 100 range. Lastly, a small portion (9%) included more than 100 PPCPs. A few methods reported a contaminants list characterized by a multiclass of compounds, not only PPCPS, but pesticides, drugs, flame retardants, etc., too. For these cases, only the total number of PPCPs was considered.

Different types of water were collected. The principal parameters monitored were temperature, pH, EC (electrical conductivity, µS cm^−1^), TDS (total dissolved solids, µg L^−1^), DO (dissolved oxygen, mg L^−1^), TSS (total suspended solids, mg L^−1^) and BOD (biochemical oxygen demand, mg L^−1^). Influent and effluent wastewater samples from hospitals or wastewater treatment plants (WWTPs) were the most analyzed (46% of studies). This matrix was marked by complexity due to the presence of numerous interferents. Some studies investigated the presence of PPCPs in raw wastewater at the treatment plant, some just the effluents, and some investigated both [35]. Most of these studies also studied the efficiency of the elimination of the PPCPs in the WWTPs [35]. All these studies identified the WWTP effluents as one source of PPCPs to the environment.

In the second block of studies, different matrices were regrouped into a single group: surface water (SW). The 40% of works analyzed and studied in this group investigated a large spectrum of water sources: streams, rivers, estuaries, lakes, seas, ground water, and urban and agricultural storm waters. Drinking and tap water (DW) constituted the third group, with an occurrence of 12% in the studies selected. DW was regulated by “The Drinking Water Directive 98/83/EC” that supervises the quality of water (for human consumption). It provided a general framework and a minimum value of 48 specific parameters that must be monitored regularly [24].

Lastly, two works included other two aqueous matrices: thermal and swimming pool water (SP). Chemicals in these matrices can come from different sources, such as bathers, who continuously release organic matter mainly through sweat and urine [25].

## 4. Conclusions and Future Trends

The review article focused on the extraction methods for PPCPs. Although these extraction methods are clearly dominated by offline solid-phase extraction using cartridges, there are significant knowledge gaps in accurately understanding the extraction mechanisms for PPCPs, including some metabolite and/or degradation products. Many of the most recent and innovative methods are based on the combination of sorbents with different chemical-physical properties either in the same cartridge, in parallel, or even in series. These modifications are considered to be small steps, but nevertheless, they represent a great advance by improving the extraction of a group of compounds with very different polarities. Multiresidue methods able to cover more than 100 compounds are already a reality. Rapid, lower cost, and eco-friendly sample preparation techniques are urgently needed. Automated, online preconcentration, and clean-up steps prior to the instrument analysis would be the future of the technique. Solvent-free microextraction methods are the trend of extractions such as DLLME. However, these techniques are rarely used, as they work especially well with non-polar PPCPs, but most are polar.

Most of the environmental studies carried out so far have two aspects, (i) analytical including validation of the methods that mainly improves the accuracy in the quantification and the elimination of the matrix effects, and (ii) the environmental aspect in which the whole cycle of the water is covered, including the identification of the sources of these compounds to the environment, the efficiency in the elimination, and the influence of environmental factors such as seasonality. All these studies have contributed to an important advance of knowledge about the distribution and hazards of PPCPs. A gap detected in these studies is the lack of knowledge about the mixtures of PPCPs found in the environment and on the different metabolites and/or degradation products that can be present. It is expected that, in the near future, there will be an increase in knowledge in these fields.

## Figures and Tables

**Table 1:** Selected applications extraction approaches to determine PPCPs in water samples.

Matrix *	No. of PPCPs	Preservation	Volume (mL)	Extraction Method	Sorbent or Cartridge	Detection	Recovery %	Reference
WW, SF	168	Na_4_EDTA	50	SPE	Clearnert PEP-2	HPLC-MS/MS	0.05–127	[32]
WW, SF	168	Na_4_EDTA	-	Direct injection	-	HPLC-MS/MS	0.05–127	[32]
SF	59	Na_2_EDTA	1000	SPE	Oasis HLB	HPLC-MS/MS	52–137	[33]
WW, SF, DW	27	-	-	SPE	Cleanert PEP	HPLC-MS/MS	74–120	[34]
WW	55	Na_2_EDTA	150	SPE	Oasis HLB	HPLC-MS/MS	9–119	[35]
SW	91	-	1000	SPE	Oasis HLB	HPLC-MS/MS	70–110	[36]
WW	12	-	7.9	DLLME	-	GC-MS/MS	91–115	[37]
WW, SW	12	-	1000	SPE	Oasis HLB	GC-MS/MS	65–115	[38]
WW, SF, DW	58	-	1.8	Online-SPE	PLRP-s	HPLC-MS/MS	70–120 (82% of total)	[39]
SW	62	-	≤20	Online-SPE	Oasis HLB	HPLC-MS/MS	81–120	[40]
SW	62	-	200	SPE	Oasis HLB	HPLC-MS/MS	81–121	[40]
WW, SW	44	Na_2_EDTA	200	SPE	Oasis HLB	HPLC-MS/MS	8–239	[41]
SW	11	-	200	SPE	Strata-X	HPLC-MS/MS	40–120	[42]
SW	34	Na_2_EDTA	400	SPE	Oasis HLB	HPLC-MS/MS	41–125	[43]
WW, SW	30	Na_2_EDTA	250	SPE	Oasis MCX	HPLC-MS/MS	78–106	[44]
SW	10	-	500	SPE	Oasis HLB	HPLC-MS/MS	69–88	[45]
WW	11	-	-	Online-SPE	TurboFlow™ column	HPLC-MS/MS	45–150	[46]
SW	16	-	10	DLLME	-	HPLC-MS/MS	70–120	[47]
WW, SW	27	Na_2_EDTA	125–500	SPE	Oasis MCX	HPLC-MS/MS	73–116	[48]
WW, SW	25 (of 41)	Na_2_EDTA	120	PES microextraction	-	HPLC-MS/MS	80–119	[49]
WW, SW	25 (of 41)	Na_2_EDTA	100–250	SPE	Oasis HLB	HPLC-MS/MS	71–131	[49]
WW, SW	10	-	20 uL	Online-SPE	Oasis HLB	HPLC-MS/MS	-	[50]
SW	12	-	500	SPE	Oasis HLB	HPLC-MS/MS	55–120	[51]
WW, SW	44	-	500	SPE innovative	GCHM, Oasis HLB	HPLC-MS/MS	76	[52]
WW	190	-	100	SPE innovative	Oasis HLB, Isolute ENV+,	UPLC-Q-TOF-MS/MS	57–120	[53]
					Strata-X-AW, Strata-X-CV			
WW	52	-	100	Disk SPE	BAKERBOND C18 Polar Plus	GC-TOF-MS	-	[54]
SW	24	Na_2_EDTA	1000	SPE	Chromabond HR-X	HPLC-MS/MS	52–117	[55]
SW	13	Na_2_EDTA	250	SPE	Strata-X	HPLC-MS/MS	51–102	[56]
SW	32	-	200	SPE	Strata-X	HPLC-MS/MS	36–119	[57]
SW	32	-	200	SPE	Strata-X-CW	HPLC-MS/MS	25–110	[57]
SP	111	-	150	SPE	Strata-X-CW	SFC-MS/MS	77 (average)	[58]
WW, SW	40	-	250	SPE	Oasis HLB	HPLC-MS/MS	17–146	[59]
WW	11	-	250	SPE	Oasis HLB	HPLC-MS/MS	53–124	[60]
SW	39	-	1000	SPE	Oasis HLB	HPLC-MS/MS	1–125	[61]
WW	15	-	250	SPE innovative	Strata-X, PSA, Alumina	GC-MS	19–103	[62]
SW	69	-	100	SPE	Strata X-CW	SFC-MS/MS	76	[63]
WW, SW	31	-	100–500	SPE	Chromabond HR-X	HPLC-MS/MS	32–97	[64]
SW	130	Na_2_EDTA	2000	SPE innovative	Oasis WAX, Oasis HLB,	HPLC-MS/MS	50–150	[65]
					Sep-Pak Plus AC 2			
WW, DW	28	-	1000	SPE	C18 Cartridges	HPLC-MS/MS	n.r.–293	[66]
WW, SW	10	Na_2_EDTA	500	SPE	Oasis HLB	UPLC-Q-TOF-MS/MS	n.r.–128	[20]
WW, SW	23	-	500	SPE	Oasis MCX	HPLC-MS/MS	54–117	[67]
WW	52	Na_2_EDTA	10	Online-SPE	Shim-pack MAYI-ODS	HPLC-MS/MS	74–104	[68]
SW	20	Na_2_EDTA	100	SPE	Strata-X	HPLC-MS/MS	70–119	[69]
WW, SW	20	-	200	SPE	Strata-X-Drug B	HPLC-MS/MS	39–102	[70]
SW	61	Na_2_EDTA	1000	Disk SPE	Speedisk^®^	HPLC-MS/MS	-	[71]
SW	61	Na_2_EDTA	200	SPE	Oasis HLB	HPLC-MS/MS	-	[71]
WW	26	Na_2_EDTA	500	SPE	Oasis HLB	HPLC-MS/MS	-	[72]
WW	10	-	-	SPE	Oasis HLB	HPLC-MS/MS	85–94	[73]
SW	35	Na_2_EDTA	1000	SPE	Oasis HLB	HPLC-MS/MS	58–194	[74]
WW, SW	20	-	300–400	SPE	Oasis HLB Prime	GC-MS	≥40%	[75]
WW	83	Na_2_EDTA	50–100	SPE	Strata-X	HPLC-MS/MS	n.r.–122	[76]
WW	59	Ascorbic acid; Na_2_EDTA	1000	SPE	Oasis HLB	HPLC-MS/MS	9–143	[77]
WW	20	Sodium thiosulfate	500	Online-SPE	Oasis HLB	HPLC-MS/MS	-	[78]
WW	20	Sodium thiosulfate	-	Direct injection	-	HPLC-MS/MS	-	[78]
SW	13	-	-	Passive sampling	PES membranes	LC-DAD	-	[79]
WW	21	-	1000	SPE	Oasis HLB	LC-HRMS	40 (average)	[80]
WW, SW	103 (of 300)	Formaldehyde	250	SPE innovative	Strata-X	UPLC- Q-TOF-MS/MS	-	[81]
WW	37	-	0.5	Online-SPE	PLRPs	HPLC-MS/MS	5–132	[82]
WW	20	-	2	Direct injection	-	HPLC-MS/MS	60–124	[83]
WW	12	-	20–100	SPE	Oasis HLB	HPLC-MS/MS	77–115	[84]
WW, SW	48	-	300–400	SPE	Oasis HLB Prime	GC-MS	>40	[85]
WW	38	-	100	SPE	Oasis MCX	HPLC-MS/MS	65–134	[86]
SW	33	-	200	SPE innovative	Oasis HLB, LC18 column	HPLC-MS/MS	50–106	[87]
SP	48	Na_4_EDTA	200	SPE	Oasis MCX	HPLC-MS/MS	71–122	[88]
WW	22	NaCl	100	Online SPE	DVB/CAR/PDMS	GC-MS	6–104	[89]
WW	19	-	250	SPE	Oasis HLB	LC-TOF/MS	5–111	[90]
WW	11	-	0.9	DLLME	-	HPLC-MS/MS	n.r.–124	[91]
WW, SW	40 (of 139)	-	1000	SPE	Oasis HLB	HPLC-MS/MS	n.r.–99	[92]
WW, SW	41 (of 139)	-	1000	SPE	Bond-Elut ENV	HPLC-MS/MS	n.r.–99	[92]
SW	10 (of 28)	-	500	Buckypaper Device	-	HPLC-MS/MS	n.r.–102	[93]
SW	44	-	200	SPE	Strata-X	HPLC-MS/MS	85–100	[94]
SW	45	Na_2_EDTA	1000	SPE	Strata-X	HPLC-MS/MS	38–112	[95]
WW	13	-	150–300	SPE	Oasis HLB	HPLC-MS/MS	40–115	[96]
SW	42	Na_2_EDTA; ASA(DW)	50	SPE	Oasis HLB	HPLC-MS/MS	33–117	[97]
WW, SW	39 (of 80)	-	500–100	SPE	Oasis MCX; Oasis HLB	HPLC-MS/MS	31–131	[98]
SW	110 (of 1153)	Phosphate buffer	1000	Disk SPE	Glass microfiber,	GC-TOF-MS/MS	-	[99]
					Empore™ SDB-XD, Empore™ AC			
WW	82	-	250	SPE	Oasis HLB	LC–Q-TOF-MS	66–149	[100]
SW	35	-	100–500	SPE	Oasis HLB	HPLC-MS/MS	2–132	[101]
WW, SW	10	-	50–100	SPE innovative	Oasis MCX, Oasis MAX	LC-HRMS	60–109	[102]
WW, SW	10	Sodium azide;	200–1000	Disk SPE	Atlantic HLB	HPLC-MS/MS	48–122	[103]
		ascorbic acid						
WW, SW	10	Sodium azide	200-1000	SPE	Oasis HLB	HPLC-MS/MS	1–110	[103]
		ascorbic acid						
WW	17	-	250	SPE	Oasis HLB	HPLC-MS/MS	<40%	[104]
SW	10	Citric acid	1000	SPE	C18	HPLC-MS/MS	97–101	[105]
WW	100	-	200	SPE	UCT XRDAH	LC-Q-TOF-MS/MS	-	[106]

* WW = Wastewater; SF = Surface water; SP = Swimming pool water. n.r. = not recovered.

**Table 2:** Mechanism, type of sorbent and target of the most used brand name of offline columns.

Brand Name	Mechanism	Sorbent	Target
Oasis HLB, HLB Prime	RP	divinylbenzene-co-*N*-vinylpyrrolidone	acidic, basic, and neutral compounds
STRATA-X	RP	styrene-divinylbenzene-co-*N*-vinylpyrrolidone	acidic, basic, and neutral compounds
Cleanert PEP	RP	divinylbenzene-co-*N*-vinylpyrrolidone-Urea	acidic, basic, and neutral compounds
Isolute ENV+	RP	polystyrene-divinylbenzene (PS-DVB)	polar compounds
Bond-Elut ENV	RP	polystyrene-divinylbenzene (PS-DVB)	polar compounds
Chromabond HR-X	RP	polystyrene-divinylbenzene (PS-DVB)	polar compounds
Oasis MCX	IC	mixed-mode CATION-exchange polymer-based	basic compounds, particularly strong bases
Oasis WAX	IC	mixed-mode ANION-exchange sorbent polymer-based	acidic compounds
Strata-X-CW	IC	mixed-mode CATION-exchange polymer-based	basic compounds, particularly strong bases
Strata-X-AW	IC	mixed-mode ANION-exchange sorbent Polymer-based	acidic compounds
Strata-X-Drug B	IC	mixed-mode strong CATION-exchange polymer-based	basic compounds, particularly strong bases
UCT XRDAH	IC	mixed-mode CATION-exchange polymer-based	basic compounds, particularly strong bases
